# SARS-CoV-2 and human retroelements: a case for molecular mimicry?

**DOI:** 10.1186/s12863-022-01040-2

**Published:** 2022-04-08

**Authors:** Benjamin Florian Koch

**Affiliations:** Department of Internal Medicine, Nephrology, Goethe University Hospital, Johann Wolfgang Goethe University Frankfurt/Main, Theodor-Stern-Kai 7, 60590 Frankfurt am Main, Germany

**Keywords:** Coronaviruses, SARS-CoV-2, COVID-19 epitope signatures, Autoimmunity, Molecular mimicry, Human retroelements, Long interspersed nuclear elements (LINE), Endogenous retroviruses (ERV)

## Abstract

**Background:**

The factors driving the late phase of COVID-19 are still poorly understood. However, autoimmunity is an evolving theme in COVID-19’s pathogenesis. Additionally, deregulation of human retroelements (RE) is found in many viral infections, and has also been reported in COVID-19.

**Results:**

Unexpectedly, coronaviruses (CoV) – including SARS-CoV-2 – harbour many RE-identical sequences (up to 35 base pairs), and some of these sequences are part of SARS-CoV-2 epitopes associated to COVID-19 severity. Furthermore, RE are expressed in healthy controls and human cells and become deregulated after SARS-CoV-2 infection, showing mainly changes in long interspersed nuclear element (LINE1) expression, but also in endogenous retroviruses.

**Conclusion:**

CoV and human RE share coding sequences, which are targeted by antibodies in COVID-19 and thus could induce an autoimmune loop by molecular mimicry.

**Supplementary Information:**

The online version contains supplementary material available at 10.1186/s12863-022-01040-2.

## Background

At the end of 2019, a severe acute respiratory syndrome (SARS)-like disease was noted in eastern China and a novel coronavirus (later designated SARS-CoV-2) recognized as the factor for the disease, COVID-19 [1]. By the spring of 2022, 447 million people have been infected globally, with 6 million casualties [2]. COVID-19 can be divided into an early viral replication phase and a late stage of organ failure [3, 4]. While the inhibition of SARS-CoV-2 replication has already been achieved [5–10], the factors driving the late phase of the disease are poorly understood [11, 12]. However, it has been reported that autoimmunity [13–27] and deregulation of human retroelements (RE) might contribute to the outcome of COVID-19 patients [28–31].

The RE share a reverse transcriptase as a common denominator. Together with an endonuclease, they can move by “copy and paste.” Based on the presence of an envelope gene, they can be divided into long terminal repeat (LTR) positive and LTR negative retrotransposons. The former and endogenous retroviruses (ERV) belong to LTR positive elements. Long interspersed nuclear elements (LINE), short interspersed nuclear elements (SINE) and SVA elements (SINE-R, VNTR and Alu) belong to LTR negative elements [32–35]. The LINE contain at least two open reading frames (ORFs), ORF1, coding for a nucleic acid binding protein with chaperone activity (ORF1p) and ORF2, which codes for a reverse transcriptase/endonuclease (ORF2p) [35, 36]. Importantly, RE make up 50 – 70% of the human genome [37, 38]. About 20% of the genome is made up from LINE sequences (c. 500,000 copies), of which more than 100 LINE1 family members are still intact and about 68 active in humans. The LINE1 show strong interpersonal differences [39, 40] and an age-dependent expression pattern [41–43]. By comparison, ERV make up about 8% of the human genome. Despite – similar to LINE – predominant inactivation, there are still hundreds of intact viral promoters and open reading frames from which the expression of ERV transcripts and proteins is possible [44–46]. The RE activation is known from many viral infections, such as HIV [47], dengue [48], influenza A [48], Zika virus [48], West Nile virus [48], measles [48], Epstein-Barr virus [49] and cytomegalovirus [50]. Therefore, I looked for the relationship of coronaviruses (CoV) to human RE based on genome, transcriptome, epitope and peptide array data. Here, transcriptome analysis coincidentally revealed many RE-identical sequences and shared epitopes in the CoV family members investigated, such as SARS-CoV-2, MERS-CoV and HKU1. To the best of my knowledge, these findings have never been reported. Importantly, epitopes are shared between human LINE1- and SARS-CoV-2 proteins and antibodies against some of these epitopes have been found to be correlated to COVID-19’s severity. In addition, RE are expressed in healthy controls and deregulated in COVID-19 patients, as well as in SARS-CoV-2-infected human cells.

## Results

The CoV genomes harbour a large number of RE-identical sequences. Several of these sequences represent shared RE-SARS-CoV-2 epitopes. Importantly, antibodies against some of these epitopes are correlated to the severity of COVID-19. In addition, RE are widely expressed in healthy controls and deregulated in COVID-19 patients, as well as in SARS-CoV-2-infected human cells.

### Sequence identity between retroelements and coronaviruses

**Fig. 1:**
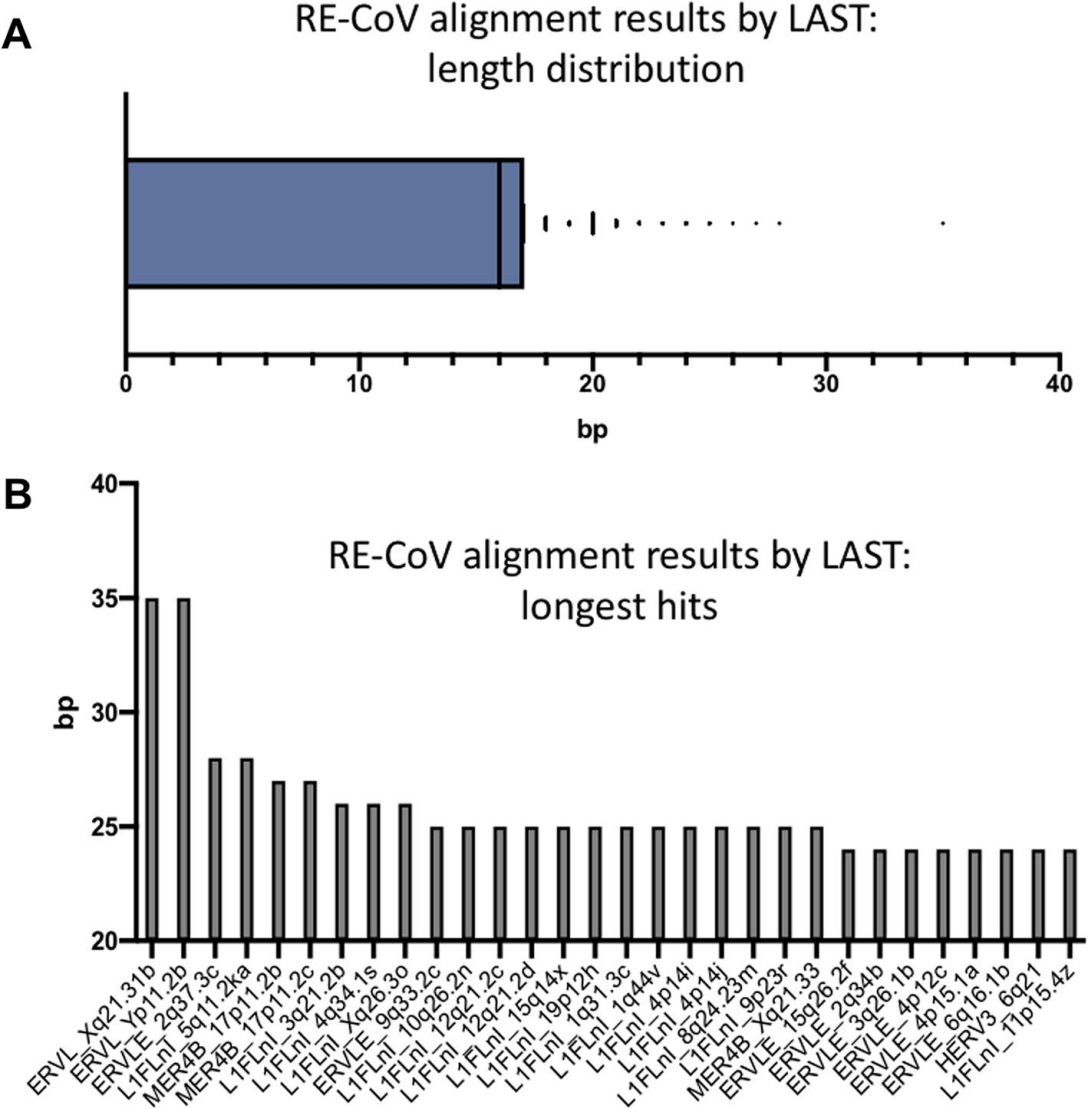
Sequence alignments of retroelements to CoV genomes by LAST. **A**. Length distribution of alignment results by LAST. **B**. Longest aligning RE-CoV sequences (LAST)

**Fig. 2:**
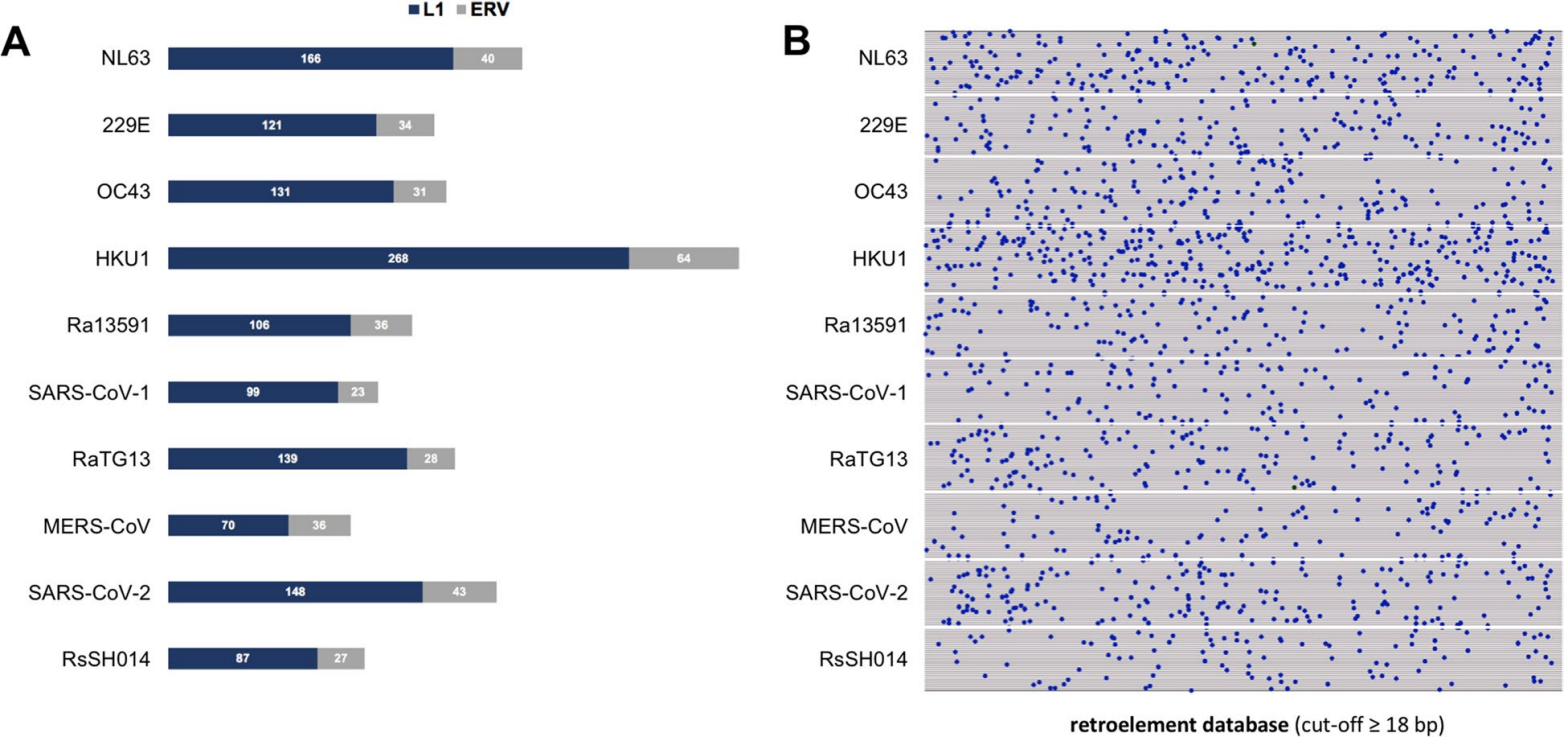
Sequence alignments of CoV genomes to retroelements by nucmer (cut-off ≥18 bp). **A**. Proportion of LINE1 (L1) and endogenous retrovirus sequences, showing a dominance of L1 sequences in all virus genomes (nucmer) analysed. **B**. Dot plot of shared RE sequences in CoV genomes, showing the highest RE-identical sequences in HKU1, followed by NL63 and SARS-CoV-2 (nucmer). Each dot represents an ≥18 bp retroelement sequence also found in the respective CoV genome

A sequence identity (≥12 bp, range 12 – 35 bp, Fig. 1A) of human RE sequences to CoV genomes from SARS-CoV-2, SARS-CoV-1, MERS-CoV, NL63, 229E, OC43, HKU1, bat CoV RA13591, bat CoV RATG13 and bat CoV RSSHC014 was found by sequence alignment of human RE sequences and different CoV genomes (Figs. 1 and 2, Table 1). Very high counts of RE-identical sequences in CoV were seen at ≥12, ≥ 15 and ≥ 18 bp (Table 1).

**Table 1:** Number of retroelement-identical sequences in CoV genomes dependent on sequence length (12 – 27 bp, based on 100% sequence identity (alignment by nucmer). Underlines indicate the highest score at the respective cut-off

CoV	Genbank ID	RE-identical sequences (nucmer)					
		≥ 12 bp	≥ 15 bp	≥ 18 bp	≥ 21 bp	≥ 24 bp	≥ 27 bp
229E	NC_002645.1	16,992	4663	**155**	8	5	4
NL63	NC_005831.2	17,751	5758	**206**	4	0	0
OC43	AY391777.1	19,428	5709	**162**	3	0	0
HKU1	NC_006577.2	19,112	6843	**332**	13	0	0
MERS-CoV	NC_019843.3	18,435	4252	**106**	3	1	0
SARS-CoV-1	AY291315.1	18,446	4731	**122**	2	1	0
SARS-CoV-2	NC_045512.2	18,917	5358	**191**	11	3	2
RsSHC014	KC881005.1	18,425	4651	**114**	5	1	0
Ra13591	MG916904.1	17,971	4644	**142**	2	0	0
RaTG13	MN996532.2	18,950	5327	**167**	4	0	0

A cut-off ≥18 bp (correlating to potential epitopes of at least 6 aa) was chosen for downstream analysis for sensitivity and epitope size reasons. A 6 aa cut-off corresponds well to a known immuno-relevant linear epitope length of 4 – 12 aa, as about 50% of them have a length ≤ 8 aa (about 25% ≤ 6 aa, and only a few of 4 aa) [51]. At this cut-off point, the majority of RE-identical sequences are seen in HKU1 (332), followed by NL63 (206) and SARS-CoV-2 (191) (Fig. 2A and B, Table 1). SARS-CoV-2 and RE sequence data were further explored by “LAST” in order to allow single nucleotide polymorphisms to be included, thereby alignments to RE sequences up to 35 bp were seen (Supplementary Table 2). In the RE-CoV data, LINE1 represent the majority of all shared sequences, while alignment to ERV sequences is a relevant minority and includes the 35 bp hits (Fig. 1B, Supplementary Tables 1 and 2). In conclusion, genome analysis revealed the presence of many short RE-identical sequences in CoV genomes, including SARS-CoV-2.

### Shared epitopes between SARS-CoV-2- and retroelement proteins

**Fig. 3:**
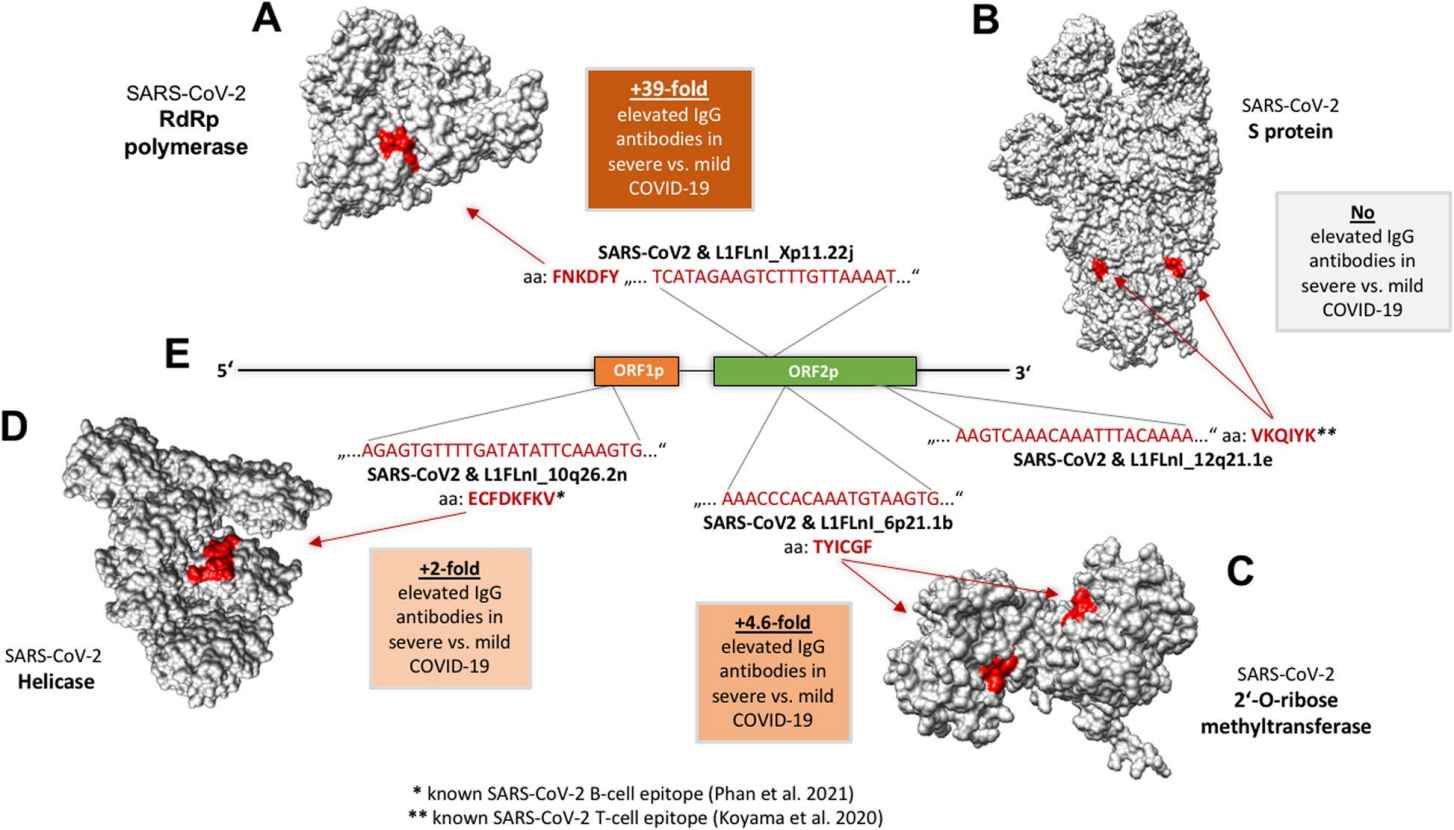
**A**. Mapping of the shared RE-CoV epitope “FNKDFY” to the SARS-CoV-2 RdRp (epitope in red), orange box depicting IgG antibody levels of severe vs. mild COVID-19 disease, with anti-FNKDFY antibodies showing 39-fold elevation in severe COVID-19. **B**. Mapping of the shared RE-CoV epitope “VKQIYK” to the SARS-CoV-2 spike protein (epitope in red), there are no reported significantly elevated antibodies against this epitope in severe COVID-19. **C**. Mapping of the shared RE-CoV epitope “TYICGF” to the SARS-CoV-2 2′-O-ribose methyltransferase (epitope in red), orange box depicting reported antibody levels of severe vs. mild COVID-19 disease, with anti-TYICGF antibodies showing a 4.6-fold elevation in severe COVID-19. **D**. Mapping of the shared RE-CoV epitope “ECFDKFKV” to the SARS-CoV-2 helicase (epitope in red). anti-ECFDKFKV antibodies showed a 2-fold elevation in severe COVID-19 **E.** Structure of a human LINE1 element with the coding regions for ORF1p (depicted in orange) and ORF2p (depicted in green)

Subsequently, all RE-identical sequences ≥18 bp were compared to the coding regions of the genome of SARS-CoV-2. Accordingly, 70 sequences showing identical aa sequences in CoV and RE were identified (Supplementary Table 1). These sequences were then compared to results from a peptide array, which investigated epitope signatures in COVID-19 patients (severe vs. mild) [52]. An overlap of human LINE1 proteins to SARS-CoV-2 epitopes from the RNA-dependent RNA polymerase (RdRp), helicase and 2′-O-ribose methyltransferase was detected for epitopes targeted with > 2-fold elevated antibody levels in severe cases (Fig. 3). Importantly, antibodies targeting an epitope of the SARS-CoV-2 RdRp polymerase, which is identical to an epitope of the LINE1 ORF2p endonuclease domain, were 39-fold elevated in severely compared to only mildly affected COVID-19 patients (Fig. 3A). The same is seen with antibodies targeting the shared CoV-RE epitopes from the 2′-O-ribose methyltransferase (Fig. 3C) and helicase (Fig. 3D). The latter is also a known B cell epitope, aa “PARARV**ECFDKFKV**” (the known B cell epitope is depicted in bold) [53]. Many other shared RE-CoV peptides (similar to those displayed in Fig. 3B) were not targeted by antibodies in severe vs. mild COVID-19 (Supplementary Table 2), but some are known as T cell epitopes, such as the one present in all three chains of the spike protein shown in Fig. 3B (aa **VKQIYK**TPPIKDF, the known T cell epitope sequence is depicted in bold) [54].

Taken together, SARS-CoV-2 and RE share peptide sequences, of which some are epitopes correlated to COVID-19 severity.

### Transcriptome analysis of retroelements in SARS-CoV-2-infected cells

**Fig. 4:**
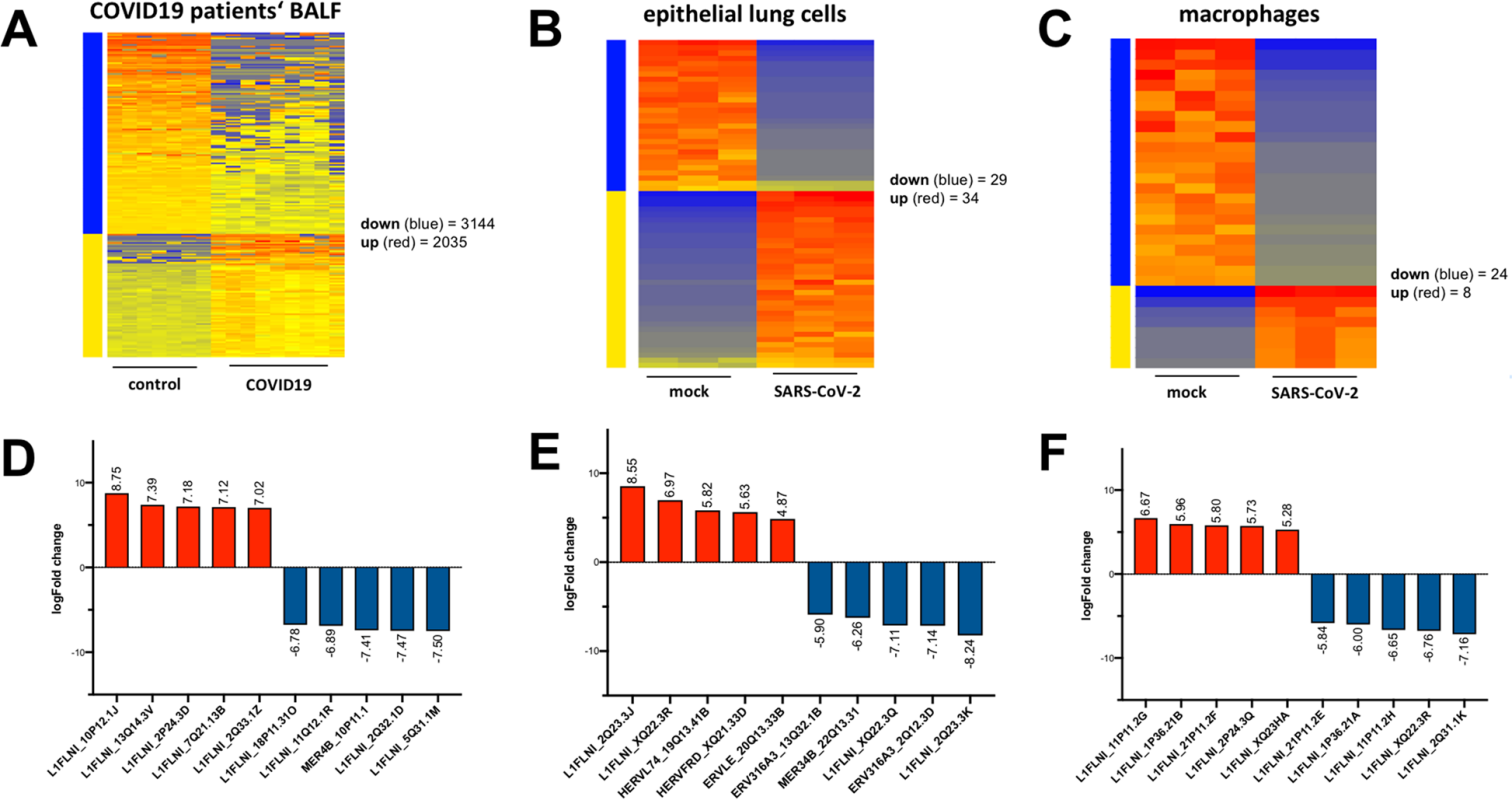
**A**. Heatmap of the most highly deregulated retroelements in bronchoalveolar lavage fluid (BALF) from COVID19 patients (red = upregulated, blue = downregulated). **B**. Heatmap of the most highly deregulated retroelements in SARS-CoV-2-infected epithelial lung cells (Calu-3). **C**. Heatmap of the most highly deregulated retroelements in SARS-CoV-2-infected macrophages. **D**. Top 10 up- and downregulated retroelements in COVID19 BALF. **E**. Top 10 up- and downregulated retroelements in SARS-CoV-2-infected epithelial lung cells. **F**. Top 10 up- and downregulated retroelements in SARS-CoV-2-infected macrophages

An RE analysis of COVID-19 patient data (bronchoalveolar lavage fluid, BALF), SARS-CoV-2 infected lung epithelial cells and SARS-CoV-2 infected macrophages was performed to explore the presence of and changes in RE expression after SARS-CoV-2 infection. Infection resulted in a highly significant (adjusted *p*-value ≤0.05) and relevant (fold change ≥2) deregulation of human RE in all samples. Transcriptome data from COVID-19 patients’ BALF compared to healthy controls shows an upregulation of 2035 and downregulation of 3144 RE (Fig. 4A). Among the top deregulated RE are mainly LINE1 (Fig. 4D). SARS-CoV-2-infected epithelial lung cells (Calu-3) show 34 up- and 29 downregulated RE (Fig. 4E), while infected human macrophages have 8 up- and 24 downregulated RE. Among the top de-regulated RE for both are also mainly LINE1 (Fig. 4E, F).

In conclusion, RE are expressed in COVID-19 patients and human cells and become deregulated after SARS-CoV-2 infection, showing mainly changes in LINE1 expression.

## Discussion

The factors driving the late phase of COVID-19 are still not fully understood [11, 12]. However, there is evidence that autoantibodies and autoreactive lymphocytes could contribute to the disease’s final outcome [13–27]. Therefore, the question of autoantibody formation in COVID-19 has to be asked. The employment of a comprehensive RE database revealed many RE-identical sequences in ten CoV family members investigated, such as in SARS-CoV-2, MERS-CoV and HKU1 (Figs. 1 and 2). Crucially, it was found that the LINE1 proteins ORF1p and ORF2p have peptides identical to SARS-CoV-2 epitopes (Fig. 3), and that some of these epitopes are associated with COVID-19’s severity, as shown by correlation to COVID-19 patients’ antibody titres (Fig. 3). In addition, RE are deregulated in COVID-19 patients (Fig. 4A), as well as SARS-CoV-2-infected human epithelial lung cells and macrophages (Fig. 4B and C), which has occasionally been reported in the last few months for cell lines and patients [28–31]. Among the analysed REs, LINE1 are strongly represented in all results (Figs. 2, 3 and 4, Supplementary Table 1 and 2). The LINE1 code for at least a nucleic acid binding protein with chaperone activity (ORF1p) and a reverse transcriptase/endonuclease (ORF2p). Importantly, autoantibodies targeting the LINE1 ORF2p endonuclease domain have been reported in 41% of SARS-CoV-1 patients [55]. The RE are also targeted by autoantibodies in several connective tissue diseases, for example, antibodies against LINE1’s ORF1p or ERV HERV-K’s envelope protein have been described in patients with systemic lupus erythematosus, lupus nephritis, rheumatoid arthritis, Sjogren’s syndrome and mixed connective tissue disease [56–65]. Relating to SARS, the autoantibodies’ target, LINE1 ORF2p, was prominently stained post-mortem in lung macrophages (residing in blood vessels), leading the authors to suspect a build-up of autoreactive CD4+ Th cells and, thus, an autoimmune loop in SARS [55]. Importantly, there is also increasing evidence for an autoimmune pathogenesis in severe COVID-19 [13–27, 66, 67]. One explanation for autoantibody formation is by molecular mimicry, i.e. shared epitopes between pathogens and hosts [68–72]. The evolution of mimicry epitopes in pathogens could be based on chance. However, although the RE-identical sequences in CoV observed are short (12 – 35 bp), the sequence lengths observed make formation by chance highly unlikely. Exemplarily, taking the genetic code (A, T, C, G) raised to a sequence of 18 bp (4^18^) results in 68,719,476,736 possible bp combinations, thus, the chance of getting one identical sequence is 1:69 billion. Additionally, a myriad of 12 bp events (Table 1) occurring by chance is stochastically very unlikely (4^12^ = 16,777,216) at more than 18,000 events. Moreover, an observed 35 bp hit such as ERVL_Xq21.31b (4^35^) corresponds to 1.18 E21 possible bp combinations, thus, the chance of getting an identical sequence is 1:1.1 trilliard – without accounting for all the other matching sequences. Therefore, recombination activities more probably account for the phenomena observed. The exchange of genetic material by recombination in RNA viruses is generally associated with virulence, host range and host response [73]. It is known that recombination in CoV can take place during co-infections at a high frequency by homologous and non-homologous recombination [74–76]. Mechanistically, an explanation could be the switching of the RdRp between multiple available RNA strands during replication [77]. This could have happened in a CoV host/ancestor with relevant LINE1 expression, as this is possible in some bat species. The black-bearded tomb bat (*Taphozous melanopogon*), for example, harbours two active LINE families [78] and shows relevant SARS-CoV-2 infection efficiency [79]. Moreover, lots of ERV families also reside in bats [80]. Therefore, serial acquisition of RE sequences, possibly taken from CoV in host animals (starting many million years ago) is a feasible scenario. Relating to the rather short sequence lengths observed, there might be an evolutionary functional constraint working against the uptake of longer RE sequences, but a benefit for the virus by coating itself with host self-antigens (“self-peptide coat”). This would dampen the innate and adaptive immune response by the presentation of “viral but self-like” peptides. The consequence of this hypothesis is in line with the view of autoimmune disease as a breakdown of self-tolerance [81, 82]. Based on the findings, autoantibodies targeting human RE could be a factor in CoV-induced disease, like COVID-19. However, this report has limitations, as the data basis for a more extensive analysis of anti-RE autoantibodies in COVID-19 still does not exist.

## Conclusion

In conclusion, it was found that CoV – including SARS-CoV-2 – harbour many RE-identical sequences, and that some of these sequences are part of SARS-CoV-2 epitopes associated with COVID-19 severity.

## Methods

### Genome analysis

Genome sequences from SARS-CoV-2 (isolate NC045512.2 = Wuhan-Hu-1), SARS-CoV-1 (AY291315.1 = FFM1), MERS-CoV (NC_019843.3 = EMC2012), human pathogenic CoVs (NC-006577.2 = HKU1; AY391777.1 = OC43, NC-002645.1 = 229E; NC-005831.2 = NL63) and bat CoVs (MN996532.2 = RaTG13, KC881005.1 = RsSHC014; MG916904.1 = Ra1359) were downloaded from GenBank (https://www.ncbi.nlm.nih.gov/genbank/). Retro.hg38.v1 (https://github.com/mlbendall/telescope_annotation_db/tree/master/builds) was employed as an RE database. The database contains 28.513 RE and is made of “RepeatMasker” hits for 60 HERV families (RepeatMasker Open-4.0, http://www.repeatmasker.org/) and all LINE elements from “L1base v2” (https://l1base.charite.de/) [83]. Alignment of the retro.hg38.v1 database to CoV genomes was done by the genome sequence aligner “nucmer” [84] (4.0.0beta2) on galaxy.org [85] and a local installation of “LAST” (v1250), a programme for genome scale sequence comparison [86]. The minimum sequence length cut-off (with 100% sequence identity) was stepwise chosen at 12, 15, 18, 21, 24, and ≥ 27, based on an immuno-relevant epitope size of about 4 – 12 amino acids (aa) (many epitopes are less than 8 aa, about 25% ≤ 6 aa, but only a few at 4 aa [51]). The nucmer “-b” and “-L” variables were used accordingly, and “Show-Coords” as well as “Mummerplot” from the “MUMmer 4” package [84] were employed to extract and plot data. Regarding to “LAST,” firstly, an RE database was built (“lastdb -uNEAR -c RE_ db retro.hg38.v1.fa”) and then CoV genomes were compared to the RE database (“lastal -D100 RE_db CoV_genome.fa > RE_db_CoV.maf”).

### Epitope-specific antibody data in COVID-19 patients

The SARS-CoV-2 epitope-specific antibody data (IgG) in severely vs. mildly affected COVID-19 patients are from Schwarz et al. [52] “Peptide microarray data – severe vs. mild – IgG,” with the peptides: 1060 (NSP12, QTVKPGNFNKDFYDF, LogFC 5.3, *p*-value 2.4E-04, FDR-adj. p-value 2.8E-02), 1243 (NSP16, ENDSKEGFFTYICGF, LogFC 2.2, p-value 4.0E-02, FDR-adj. p-value 5.2E-01), 1227 (NSP13, IPARARVECFDKFKV, LogFC − 0.9, p-value 3.2E-01, FDR-adj. p-value 5.3E-01) and 1690 (Spike, AQVKQIYKTPPIKDF, LogFC 0.2, p-value 8.3E-01, FDR-adj. p-value 8.5E-01). “L1base v2” was used for comparison with coding LINE1 sequences (https://l1base.charite.de/) [83]. Known SARS-CoV-2 B- and T-cell epitopes are from Phan et al. [53] and Griffoni et al. [54]. The PDB data for the SARS-CoV-2 RdRp (PDB ID: 7BW4), helicase (PDB ID: 7NNG), 2′-O-ribose methyltransferase (PDB ID: 7JYY) and -spike protein (PDB ID: 7LSS) were downloaded from https://www.rcsb.org and epitopes displayed by “UCSF Chimera v1.15” (for Mac OS) [87].

### Transcriptome analysis

Total RNA sequencing data from SARS-CoV-2-infected macrophages (BioProject ID PRJNA637580, Sequence Read Archive (SRA) ID mock: SRR11934391, SRR11934392, SRR11934393, infected: SRR11934394, SRR11934395, SRR11934396) [88], Calu-3 adrenocarcinomic lung epithelial cells (PRJNA615032, mock: SRR11517744, SRR11517745, SRR11517746, infected: SRR11517747, SRR11517748, SRR11517749) [89] and bronchoalveolar lavage (BALF) samples from intensive care COVID-19 patients (PRJNA605983SRA, SRA: SRR11092056, SRR11092057, SRR11092058, SRR11092059, SRR11092060, SRR11092061, SRR11092062, SRR11092063, SRR11092064) [90] compared to healthy controls (PRJNA316136, SRA: SRR3286988, SRR3286989, SRR3286990, SRR3286991, SRR5515942, SRR5515943, SRR5515944) [91] were downloaded from SRA (https://www.ncbi.nlm.nih.gov/sra), quality controlled by FastQC (Babraham Institute, Cambridge, UK, http://www.bioinformatics.babraham.ac.uk/projects/fastqc/) and Illumina adapters trimmed by Trimmomatic [92]. Salmon [93] and DESeq2 [94] were employed for differential RE analysis, with standard parameters after indexing the retro.hg38.v1 database (“salmon index -t retro.hg38.v1.fa -i retro.hg38.v1_index -k 31”). Heatmaps were done by iDEP v0.92 [95] and graphs by GraphPad Prism software version 8.0 for OS X (GraphPad Software Inc., USA).

## Supplementary Information

**Additional file 1: Supplementary Table 1.** RE – CoV sequence alignment results by nucmer.

**Additional file 2: Supplementary Table 2.** RE – CoV sequence alignment results by LAST.

## Data Availability

All data generated in this study are included in this article and its supplementary files. The data used in this study are openly available at the sources detailed in the methods section.

## Abbreviations

229E: Human coronavirus 229E
aa: Amino acids
BALF: Bronchoalveolar lavage
bp: Base pairs
CoV: Coronaviruses
COVID-19: Coronavirus disease 2019
ERV: Endogenous retroviruses
HKU1: Human coronavirus HKU1
LINE1: Long interspersed nuclear elements
LTR: Long terminal repeat
MERS-CoV: Middle East respiratory syndrome-related coronavirus
NL63: Human coronavirus NL63
OC43: Human coronavirus OC43
ORF: Open reading frame
Ra1359: Bat coronavirus Ra1359
RaTG13: Bat coronavirus RaTG13
RdRp: RNA-dependent RNA polymerase
RE: Human retroelements
RsSHC014: Bat coronavirus RsSHC014
SARS-CoV-1: Severe acute respiratory syndrome coronavirus type 1
SARS-CoV-2: Severe acute respiratory syndrome coronavirus type 2
SINE: Short interspersed nuclear elements
SRA: Sequence read archive
SVA: SINE-R, VNTR and Alu
